# Genetic Variation among Temporally and Geographically Distinct West Nile Virus Isolates, United States, 2001, 2002

**DOI:** 10.3201/eid0911.030301

**Published:** 2003-11

**Authors:** C. Todd Davis, David W.C. Beasley, Hilda Guzman, Pushker Raj, Mary D’Anton, Robert J. Novak, Thomas R. Unnasch, Robert B. Tesh, Alan D.T. Barrett

**Affiliations:** * Center for Biodefense and Emerging Diseases, Galveston, Texas, USA;; † University of Texas Medical Branch, Galveston, Texas, USA;; ‡ Texas Department of Health-Rabies/Arbovirus Section, Austin, Texas, USA;; § Illinois Natural History Survey, Champaign, Illinois, USA;; ¶ University of Alabama at Birmingham, Birmingham, Alabama, USA

**Keywords:** West Nile virus, flavivirus, encephalitis, molecular epidemiology, phylogenetics, geographic distribution, viral evolution, nucleotide sequencing, genetic variation, emerging disease

## Abstract

Analysis of partial nucleotide sequences of 22 West Nile virus (WNV) isolates collected during the summer and fall of 2001 and 2002 indicated genetic variation among strains circulating in geographically distinct regions of the United States and continued divergence from isolates collected in the northeastern United States during 1999 and 2000. Sequence analysis of a 2,004-nucleotide region showed that 14 isolates shared two nucleotide mutations and one amino acid substitution when they were compared with the prototype WN-NY99 strain, with 10 of these isolates sharing an additional nucleotide mutation. In comparison, isolates collected from coastal regions of southeast Texas shared the following differences from WN-NY99: five nucleotide mutations and one amino acid substitution. The maximum nucleotide divergence of the 22 isolates from WN-NY99 was 0.35% (mean = 0.18%). These results show the geographic clustering of genetically similar WNV isolates and the possible emergence of a dominant variant circulating across much of the United States during 2002.

West Nile virus (WNV) is a member of the genus *Flavivirus* (family *Flaviviridae*) and belongs to the Japanese encephalitis virus serocomplex. Until 1999, the geographic distribution of the virus was limited to Africa, the Middle East, India, and western and central Asia with occasional epidemics in Europe (1,2). By December 2002, however, the distribution of the virus had expanded to include 44 states of the continental United States and southern regions of 5 Canadian provinces from Saskatchewan to Nova Scotia (3). Over the course of 3 years, the virus has traversed North America, presumably from New York City, where it was first isolated during the summer of 1999 (4–7). Partial nucleotide and complete genome sequence analysis of several WNV strains isolated in the northeastern United States during 1999 and 2000 showed that these isolates were most closely related to a WNV strain isolated from the brain of a dead goose in Israel in 1998 (6,8,9). The subsequent establishment of WNV across the eastern and midwestern regions of North America from 1999 through 2001 set the stage for the rapid and widespread movement of the virus across the remainder of the continent during the summer of 2002, resulting in the highest number of annual case reports and deaths attributed to WNV in humans, equines, and birds documented since the discovery of the virus in North America. Surveillance programs initiated by public health agencies, research institutions, and diagnostic laboratories have resulted in the collection of hundreds of WNV isolates across the United States and Canada from various sources, including mosquitoes, humans, equines, birds, and a number of other vertebrate species (3).

Phylogenetic comparisons of partial and complete nucleotide sequences from isolates collected in the northeastern United States during 1999 and 2000 demonstrated a high degree of genetic similarity to the prototype New York strain, WN-NY99 (GenBank accession no. AF196835), with nucleotide identities of >99.8% and amino acid identities of >99.9% (9–12). Although these studies have confirmed that northeastern isolates collected in 1999 and 2000 showed limited genetic divergence from WN-NY99, to date little published information has described the continuing divergence of WNV as its temporal and spatial distribution have expanded (13). To assess the extent to which WNV has evolved since its introduction in North America, we analyzed the partial nucleotide and deduced amino acid sequences of WNV isolates collected during the summer and fall of 2001 and 2002 and compared them to a homologous sequence region of WN-NY99. Collaborations between the University of Texas Medical Branch (UTMB) and a number of U.S. public health agencies have allowed 22 isolates of WNV to be collected, representing several geographically distinct U.S. regions. Phylogenetic comparisons of a 2,004-nucleotide region encoding the entire premembrane and envelope proteins (prM-E) of each isolate have shown the most divergent variants of WNV in North America to date and provide evidence of the possible emergence of a dominant variant circulating in many regions of the United States. Furthermore, our results indicate geographic clustering of distinct variants within and between states and reinforce previous evidence supporting the likelihood of multiple introductions of virus into the state of Texas (13).

## Materials and Methods

### Collection and Virus Isolation

**Figure 1:**
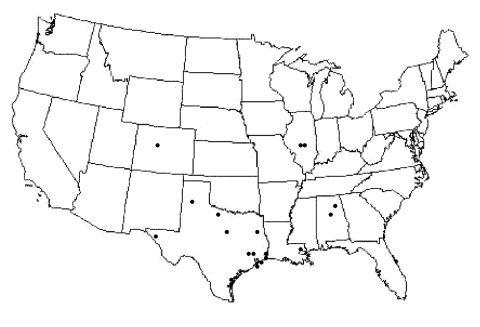
Locations of collected isolates, 2001–2002.

Isolates were collected from five states: Illinois, Alabama, Louisiana, Colorado, and Texas. Isolates from Texas were collected from nine counties representing regions across the entire state (Figure 1). All isolates were collected from September 2001 to October 2002. After being confirmed WNV-positive by state public health laboratories, virus or tissues were sent to UTMB for submission into the World Arbovirus Reference Collection. Each sample was given one passage in Vero cells to derive viruses for use in these studies. Virus samples represented a variety of sources, including mosquito pools, bird brain, human cerebrospinal fluid (CSF), and a dog kidney. Of the 18 isolates sequenced in this study (Tables 1 and 2), 11 were isolated from mosquito pools by the Texas Department of Health (TDH); 2 from a mosquito pool and dog kidney homogenate by the Illinois Natural History Survey (INHS); 2 from passerine brain homogenates from the University of Alabama at Birmingham; 1 from a red-tailed hawk brain homogenate by the Centers for Disease Control and Prevention, Division of Vector-Borne Infectious Diseases (CDC-DVBID), Fort Collins, Colorado; 1 from a mosquito pool in Louisiana, courtesy of CDC-DVBID; and 1 from the CSF of a patient who died of West Nile encephalitis at UTMB.

**Table 1:** Nucleotide mutations in sequences of the prM gene of 22 West Nile virus isolates obtained during 2001 and 2002 compared to WN-NY99^a^

Strain	Source	Collected	RNA origin	prM (501 nt)								
				491 (prM9)	507	549	621	660	679 (prM72)	690	807	903
WN-NY99 (AF196835)	Flamingo	06/01/99	Brain	A (Lys)	A	U	A	C	U (Ser)	C	C	G
Harris Co., TX (AY185906)	Bluejay	06/11/02	Brain/Vero					U				
Harris Co., TX (AY185907)	Bluejay	06/10/02	Brain/Vero					U				
Nueces Co., TX - 1	*Culex* *quinquefasciatus*	08/06/02	BHK	G (Arg)				U				
Nueces Co., TX - 2	*Culex* *quinquefasciatus*	09/17/02	BHK					U				
Gregg Co., TX	*Culex* *quinquefasciatus*	09/25/02	BHK					U				
Tarrant Co., TX	*Culex* *restuans*	09/30/02	Vero									
Wichita Co., TX	*Culex* *quinquefasciatus*	10/23/02	BHK					U				
Randall Co., TX	*Culex* *tarsalis*	09/18/02	BHK					U				
El Paso Co., TX	*Culex* *tarsalis*	08/26/02	Vero					U				
Illinois - 1	*Culex pipiens*	08/02/02	Vero			A				U		
Illinois - 2	Dog	08/01/02	Kidney/Vero		G			U				
Alabama - 1	*Culex* *quinquefasciatus*	10/05/01	Vero									
Alabama -2	Crow	09/10/01	Brain/Vero									
Colorado	Red-tailed Hawk	08/01/02	Brain/Vero					U				
Louisiana	*Culex salinarius*	08/06/02	Vero								U	
Galveston Co., TX-1 (AY185914)	Bluejay	08/02/02	Vero				G		A (Thr)			
Galveston Co., TX-2 (AY185913)	Bluejay	07/19/02	Vero						A			
Galveston Co., TX-3	*Culex* *quinquefasciatus*	08/21/02	Vero									
Jefferson Co., TX - 1	*Culex* *quinquefasciatus*	08/06/02	BHK									
Jefferson Co., TX - 2	*Culex* *quinquefasciatus*	07/02/02	BHK									A
Jefferson Co., TX - 3	Human	08/24/02	CSF/Vero									
Orange Co., TX	*Culex* *quinquefasciatus*	07/03/02	Vero									A

*Nucleotide (nt) numbers correspond to those of WN-NY99 (GenBank accession no. AF196835); in brackets for individual residues are the deduced amino acid changes.f

**Table 2:** Nucleotide mutations in sequences of the E gene of 22 West Nile virus isolates obtained during 2001 and 2002 compared to WN-NY99^a^

Strain	Envelope (1503 ntds)																							
	969	1038	1065	1071	1118 (E51)	1137	1179 (E71)	1192 (E76)	1293	1356	1377	1442 (E159)	1443	1554	1557	1581	1728	1830	2094	2154	2190	2392 (E476)	2400	2466
WN-NY99 (AF196835)	C	U	C	U	C (Ala)	C	A (Lys)	A (Thr)	C	C	C	U (Val)	U	T	C	T	A	T	A	U	U	G (Ala)	U	C
Harris Co., TX (AY185906)												C										A (Thr)		U
Harris Co., TX (AY185907)			U			U						C												U
Nueces Co., TX - 1											U	C									C			U
Nueces Co., TX - 2					U (Val)				U		U	C												U
Gregg Co., TX												C	C											U
Tarrant Co., TX		C										C												U
Wichita Co., TX												C												U
Randall Co., TX					U		C (Asn)					C												U
El Paso Co., TX												C												U
Illinois - 1												C												U
Illinois - 2												C			U	C		C						U
Alabama - 1												C												U
Alabama -2												C					T							U
Colorado												C							G					U
Louisiana																								
Galveston Co., TX-1 (AY185914)	U							G (Ala)		U										C			C	
Galveston Co., TX-2 (AY185913)	U							G		U										C			C	
Galveston Co., TX-3	U							G		U				C						C			C	
Jefferson Co., TX - 1	U							G		U										C			C	
Jefferson Co., TX - 2	U			C				G		U										C			C	
Jefferson Co., TX - 3	U							G		U										C			C	
Orange Co., TX	U			C				G		U										C			C	

*Nucleotide (nt) numbers correspond to those of WN-NY99 (GenBank accession no. AF196835); in brackets for individual residues are the deduced amino acid changes.f

### RNA Extraction, Reverse Transcription, and Polymerase Chain Reaction

Viral RNA was extracted directly from 140 mL of infected Vero or BHK cell culture supernatants by using the QiaAMP viral RNA extraction kit (Qiagen, Valencia, CA). Reverse transcription (RT) was performed in a 50-mL volume containing 5 mL of viral RNA, 1 mL of random hexamer primer, 10 mL of 5X RT buffer, 4 mL of 10 mM dNTPs, 0.4 mL of cloned RNAse inhibitor, 0.5 mL of Moloney murine leukemia virus (MMLV) reverse transcriptase, and 29.1 mL of high-performance liquid chromatography (HPLC) water. Polymerase chain reaction (PCR) was performed in a 25-mL volume containing 2.0 mL cDNA template from RT, 1.0 mL forward primer, 1.0 mL reverse primer, 2.5 mL 10X PCR buffer, 0.5 mL 10 mM dNTPs, 0.5 mL of 1 U/mL Taq PCR, and 17.5 mL of HPLC water. Three previously described primer pairs were used to amplify the entire prM-E genes of WNV (13). PCR products were gel-purified by using the QIAquick kit (Qiagen), according to the manufacturer’s protocol, and the resulting template was directly sequenced by using the amplifying primers. The WN1751/WN2504A PCR product derived from WNV isolate Galveston County, TX-3 was cloned into pGEM-T Easy (Promega Corporation, Madison, WI), and 10 clones were sequenced to determine the degree of nucleotide sequence divergence within a single isolate collected from the southeast coast of Texas. Sequencing reactions were performed in the UTMB Biomolecular Resource Facility’s DNA sequencing laboratory by previously described methods (13). Analysis and assembly of sequencing data were performed by using the Vector NTI Suite software package (Informax, Frederick, MD). Nucleotide and deduced amino acid sequences of the entire prM-E genes from each isolate were aligned by using the AlignX program in the Vector NTI Suite and compared with previously published sequences of isolates from southeast Texas collected from June to August of 2002 (13). All isolates were then compared with isolates collected in the northeastern United States during 1999, 2000, and 2001, and a phylogenetic tree was constructed by maximum parsimony algorithm by using PAUP (Version 4.0b10) (Sinauer Associates, Sunderland, MA) to show genetic relationships of these isolates with other North American WNV isolates found in GenBank, in which the homologous 2,004-nucleotide region had been sequenced.

## Results

Nucleotide sequences representing a 2,004-nucleotide region of the complete prM-E genes of WNV (nucleotides 466–2,469) of the 18 isolates collected in 2001 and 2002 (GenBank accession nos. AY4281514-AY428531), plus 4 southeast Texas strains (13), were compared with a homologous sequence region of the prototype WNV, WN-NY99 (Table 1). Of the 22 isolates analyzed, 16 were collected from 10 different Texas counties, and 2 each from Illinois and Alabama, plus 1 each from Colorado and Louisiana. All isolates were from 2002, except 2 that came from Alabama in 2001 (Figure 1). Sequence alignments comparing WN-NY99 with individual 2001 and 2002 isolates showed up to seven nucleotide mutations and three amino acid substitutions among the 22 isolates analyzed (Table 1). Nucleotide mutations occurred at 33 positions (9 in prM, 24 in E) with a total of 7 amino acid substitutions (2 in prM, 5 in E). The maximum nucleotide divergence of the 22 isolates from WN-NY99 was 0.35%, with an average nucleotide divergence of 0.18%.

**Figure 2:**
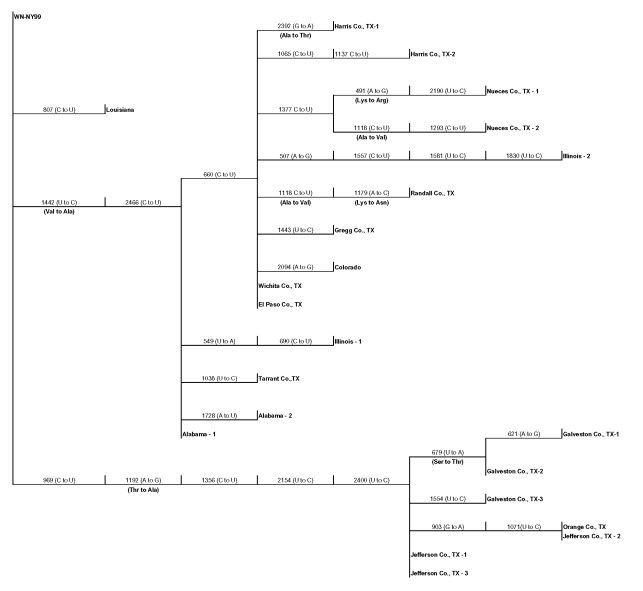
Phylogram based on maximum parsimony analysis comparing a 2,004-nucleotide sequence of WN-NY99 (GenBank accession no. AF196835) with 22 West Nile virus asolates collected during 2001 and 2002.

**Figure 3:**
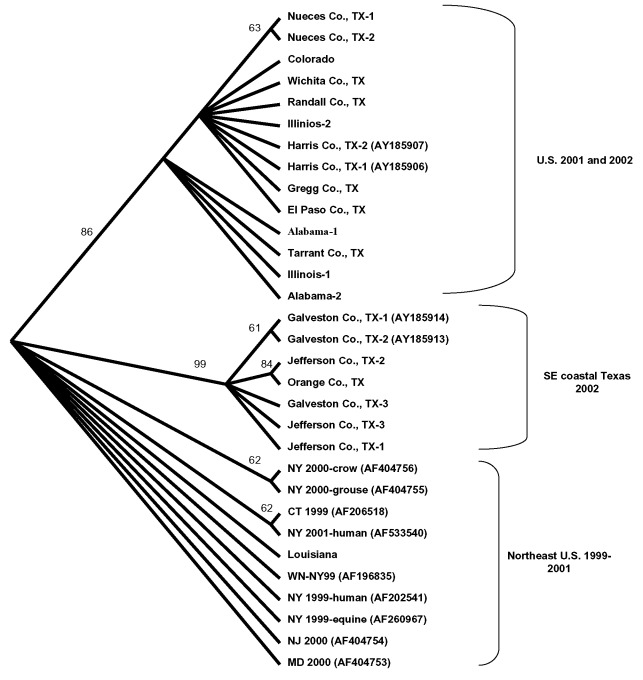
Cladogram based on maximum parsimony analysis comparing a 2,004-nucleotide sequence of 22 West Nile virus isolates collected during 2001 and 2002 with a homologous region of WN virus isolates collected in 1999, 2000, and 2001 from the northeastern United States. Numbers indicate bootstrap confidence estimates based on 500 replicates for clades supported to the right. Numbers in parenthesis represent GenBank accession numbers.

Several of the nucleotide mutations identified in this study were shared by many isolates (Tables 1 and 2; Figure 2). Two nucleotide mutations at residues 1,442 (conservative amino acid substitution of Val to Ala at position E159) and 2,466 were shared by 14 of the 22 isolates, with 10 of these 14 isolates sharing an additional noncoding nucleotide mutation at residue 660. Five different nucleotide mutations (at residues 969, 1,192 [amino acid substitution of Thr to Ala at position E76], 1,356, 2,154, and 2,400) were shared by seven isolates, all of which were collected from coastal regions of southeast Texas. The isolate from Louisiana differed from WN-NY99 at only one nucleotide (residue 807) over the region studied and did not share any nucleotide mutations with other isolates from this study. In comparison, all other nucleotide mutations identified in this study were not shared by nucleotide sequences reported previously from isolates collected in the northeastern United States during 1999, 2000, or 2001 (9–12). Because these mutations were unique to isolates sequenced during this study, our results did not show a closer genetic relationship to isolates from 2001, 2000, or 1999. However, the two isolates in this study that were collected in 2001 (Alabama-1; Alabama-2) did share two nucleotide mutations (residues 1,442 and 2,466) with 12 of the other isolates collected in 2002. Construction of a phylogenetic tree by maximum parsimony analysis (Figure 3) illustrates the genetic proximity of isolates from this study to those collected from the northeastern United States in 1999, 2000, and 2001. Branch groupings showed both temporal and geographic separation of isolates, with those collected in the northeastern United States in 1999, 2000, and 2001 representing a distinct clade in relation to isolates collected in 2002. An exception to this grouping is an isolate from Louisiana collected in 2002, which was grouped with northeastern United States isolates from 1999 to 2001. Notably, WNV isolates from the southeastern coast of Texas also comprise a clade of their own, separating these isolates from other 2001 and 2002 isolates collected from various regions within the United States. A recently reported WNV isolate collected from a Missouri dog in 2002 (GenBank accession no. AY160126) also shared a nucleotide mutation (residue 2,466 C to U) with the 2002 isolates from this study. Although the entire prM-E gene of this isolate was not reported, this isolate likely represents an additional member of the large 2002 clade.

In a previous report concerning the genetic divergence of WNV since its introduction into the United States, Beasley et al. (13) described a quasispecies population within a single WNV isolate from Harris County, Texas. To determine whether nucleotide mutations that define the southeast coastal Texas variant were uniform throughout the quasispecies population of a select isolate, the WN1751/WN2504A PCR product derived from WNV isolate Galveston Co., TX-3, was cloned into pGEM-T Easy. Ten clones were sequenced to obtain homologous regions of 700 nucleotides, which were then compared with the Galveston Co., TX-3, consensus sequence. This region contained the U to C mutation at nucleotide 2154 and the U to C mutation at nucleotide 2,400. Five of the 10 clones were identical to the consensus sequence, while the other five clones each had one or two nucleotide changes from the consensus sequence for a total of eight nucleotide changes (Table 3). None of the mutations identified represented amino acid substitutions and, unlike the 2001–2002 variant population (13), none of the mutations encoded a stop codon. The maximum nucleotide divergence of individual clones was 0.28% (mean = 0.11%). Furthermore, none of the nucleotide changes identified in the five clones was shared with WNV strains representing the 2001–2002 variant, nor were any nucleotide changes identified at two of the nucleotide positions that defined the southeastern coastal Texas variant. These results suggest that none of the virus genomes existing in a quasispecies population from WNV isolate Galveston Co., TX-3, contained nucleotide mutations characteristic of the 2001–2002 variant identified in this study.

**Table 3:** Nucleotides that varied among individual clone sequences of a fragment of the E protein gene (genomic positions 1,769-2,469) of the WNV Galveston Co., TX-3^a,b^

Clone	Nucleotide							
	1,779	1,787	1,798	1,871	2,162	2,168	2,232	2,469
Consensus sequence	U	U	A	A	A	G	A	U
1			G				G	
2	C	C						
4				G				C
6					G			
7						A		

^a^WNV, West Nile virus. ^b^Clones 3, 5, 8, 9, 10 are identical to the consensus sequence.

## Discussion

Sequence comparisons of a 2,004-nucleotide region of 22 WNV isolates collected during the summer and fall of 2001 and 2002 showed the highest degree of nucleotide divergence from WN-NY99 to date. Studies by Lanciotti et al. (9) and Huang et al. (12) have shown that the complete genomes of several WNV isolates collected in 1999, 2000, and 2001 share >99.8% nucleotide identity with WN-NY99, with three or fewer amino acid substitutions in the entire polyprotein. Similar studies of partial nucleotide sequences conducted by Anderson et al. (10) and Ebel et al. (11) reported up to three nucleotide mutations encompassing a region of 921 nucleotides and 1,503 nucleotides from isolates collected in Connecticut in 1999 and 2000 and New York in 2000, respectively. Although our studies have compared a larger portion of the genome than earlier studies of partial nucleotide sequences, we have identified individual isolates with as many as seven nucleotide mutations and three amino acid substitutions, with a maximum divergence of 0.35% from the homologous region of the prototype North American WNV, WN-NY99. The nucleotide mutations identified in this study were not shared by previously sequenced isolates from 1999, 2000, or 2001 (9–12) and represent new nucleotide changes in the North American WNV population. Since these changes were not shared with other previously reported WNV sequences, the isolates analyzed in this study did not show a greater genetic similarity with northeastern isolates from 1999, 2000, or 2001. However, several of these nucleotide changes (660, 969, 1,356, 2,154, 2,400, and 2,466) are observed in other Old World WNV strains from both lineage I and lineage II (Table 4). Each of these changes represents a noncoding mutation from either a C to U or U to C in the third codon of the open reading frame; nucleotides at these positions may revert back to nucleotides observed in the more ancestral Old World strains.

**Table 4:** Nucleotide changes from WN-NY99 observed in 2001 and 2002 WNV isolates that are conserved in Old World WNV isolates with complete genomes available from Genbank^a,b^

660 (C to U)	969 (C to U)	1,356 (C to U)	2,154 (U to C)	2,400 (U to C)	2,466 (C to U)
WN Uganda 1937 (M12294)	WN IS-98 STD (AF481864)	WN Eg101	WN Uganda 1937	WN Uganda 1937	WN Uganda 1937
WN LEIV-Krnd88-190 (AY277251)	WN Eg101 (AF260968)			WN LEIV-Krnd88-190	
	WN Ast99-901 (AY278441)			WN Eg101	
	WN LEIV-Krnd88-190			WN Ast99-901	
				WN RO97-50 (AF260969)	
				WN VLG-4 (AF317203)	
				WN KN3829 (AY262283)	
				WN Italy 1998-equine (AF404757)	
				WN LEIV-Vlg00-27924 (AY278442)	
				WN VLG-4 (AF317203)	

^a^WNV, West Nile virus. ^b^Number in parenthesis represents GenBank accession no.

Our results also suggest the geographic clustering of genetically distinct variants. Seven of the 22 isolates, all of which were collected from coastal regions of southeast Texas, share five nucleotide mutations unique to only these isolates. Fourteen of the other isolates, which represent the CDC-defined East South Central (AL), West South Central (LA and TX), East North Central (IL), and Mountain (CO) regions (3), all share two unique nucleotide mutations not identified in other isolates (Figure 2). The results of this study support the findings of Beasley et al. (13), which suggest that during the summer of 2002 WNV was introduced into Texas on at least two separate occasions. These results might reflect the unique migratory patterns of North American birds, which act as reservoir hosts for WNV. As Rappole et al. (14) have illustrated, many North American birds follow well-documented migration routes from summer grounds in the northeastern United States to southern areas that are classified as the southeastern United States, circum-Gulf, trans-Gulf, and Caribbean/western North Atlantic routes. For example, the Laughing Gull (*Larus atricilla*) has been known to follow a circum-Gulf route as it travels from the northeastern United States to stopover sites along the northern and western Gulf Coast on its way to Mexico or Central America. Because certain species of birds have a more limited geographic range than others, geographically clustered populations of distinct genetic variants, for example, isolates collected from coastal regions of southeast Texas, might arise as a result of restricted migratory routes. This hypothesis is supported by a number of studies. Peiris and Amerasinghe (15) have identified a group of geographically restricted antigenic variants of WNV confined to southern India. Because of the lack of bird migratory routes linking southern India with the Middle East and Africa, a distinct antigenic group exists exclusively in southern India. Furthermore, numerous studies have shown antigenic variation among WNV strains that correlate with geographically distinct regions and restricted bird migratory patterns (16,17). Phylogenetic comparisons of Indian viruses with other WNV strains show similar findings, which place Indian WNV strains in a unique clade of lineage I (9,18). Recent studies in Israel by Malkinson et al. (19) also support the role of migratory birds in the dispersion of unique WNV variants in geographically distinct regions. The results of our study support an alternative hypothesis that explains the continental spread of WNV as a consequence of transmission between local bird and mosquito populations in a given region. This mechanism allows for spread of the virus from region to region over shorter distances, in contrast to the long distances traveled by migratory birds (20). Our finding of a dominant variant that exists over a large part of the United States, together with evidence of a geographically distinct southeast coastal Texas variant, suggests that both mechanisms of spread have influenced the genetic distribution and spread of WNV in the United States.

To date, little genetic evidence supports or refutes the hypothesis that WNV becomes established in an enzootic transmission cycle in a particular geographic area rather than being reintroduced into a particular area each year when the transmission season begins. Similarly, because of the limited published data detailing the year-to-year genetic changes observed in WNV, whether the virus is becoming endemic in particular regions of the United States remains to be established. This question will be answered in part by determining baseline phylogenetic results of specific variants in a geographic area and by analyzing isolates collected in sequential transmission seasons.

Although the isolates analyzed in this study do not represent the entire temporal and geographic distribution of WNV in North America, at least some nucleotide mutations have been conserved among WNV strains circulating across the continent. If indeed the conservation of these mutations is the result of selective pressure, such as the continued capacity to replicate in both arthropod and vertebrate hosts, rather than random mutations occurring as a consequence of genetic drift, one would expect these mutations to be conserved in virus isolates collected in other regions of North America. Further investigation concerning the genetic composition of viruses from additional regions of North America will define the extent to which dominant variants have emerged. If dominant variants do continue to emerge across the United States, phylogenetic analyses will help researchers monitor the spread of WNV in North America and may provide explanations for the rapid and widespread movement of this newly emerging virus in North America. Similarly, identifying the genetic composition of WNV isolates from other regions of the United States and Canada, as well as comparing these isolates with isolates collected in 2003, will continue to define evolutionary relationships of WNV circulating in North America and facilitate predictions concerning the primary mechanisms of transmission and spread of the virus.
