# Correction Vol. 9, No. 11

**DOI:** 10.3201/eid1001.C21001

**Published:** 2004-01

**Keywords:** errata, erratum, correction

In “Genetic Variation among Temporally and Geographically Distinct West Nile Virus Isolates, United States, 2001, 2002”, by C. Todd Davis, et al., errors occurred in Tables 1 and 2. The correct title for Table 1 is “Nucleotide mutations in sequences of the prM gene of 22 West Nile virus isolates obtained during 2001 and 2002 compared to WN-NY99.” In Table 1, line 6, Nueces Co., Tx-1, under “prM 491” should read “G (Arg)”; line 19, Galveston Co, TX-2, under “prM 679” should read “A (Thr).” The correct title for Table 2 is “Nucleotide mutations in sequences of the E gene of 22 West Nile virus isolates obtained during 2001 and 2001 compared to WNNY99.”

In Table 2, line 3, Harris Co., TX, under “Envelope 2,392,” should read “A (Thr)”; line 8, Nueces Co., TX-2, under “Envelope 1,118,” should read “U (Val)”; line 12, Randall Co., TX, under “1,192” should read “C (Asn)”; line 24, Galveston Co., TX-3, under “Envelope 1,192” should read “G (Ala).” Corrected versions of Tables 1 and 2 are available at: http://wwwnc.cdc.gov/eid/article/9/11/03-0301_article.htm.

We regret any confusion these errors may have caused.

